# Flexible Electrochromic Device on Polycarbonate Substrate with PEDOT:PSS and Color-Neutral TiO_2_ as Ion Storage Layer

**DOI:** 10.3390/polym15091982

**Published:** 2023-04-22

**Authors:** Christopher Johannes, Sven Macher, Lukas Niklaus, Marco Schott, Hartmut Hillmer, Michael Hartung, Hans-Peter Heim

**Affiliations:** 1 Plastics Engineering, Institute of Material Engineering, University of Kassel, 34125 Kassel, Germany; 2 Fraunhofer Institute for Silicate Research ISC, Neunerplatz 2, 97082 Würzburg, Germany; 3 Technological Electronics, Institute of Nanostructure Technologies and Analytics, University of Kassel, 34125 Kassel, Germany

**Keywords:** flexible electrochromic device, smart windows, PEDOT:PSS, titanium dioxide, polyurethane-based electrolyte

## Abstract

Electrochromic (EC) windows on glass for thermal and glare protection in buildings, often referred to as smart (dimmable) windows, are commercially available, along with rearview mirrors or windows in aircraft cabins. Plastic-based applications, such as ski goggles, visors and car windows, that require lightweight, three-dimensional (3D) geometry and high-throughput manufacturing are still under development. To produce such EC devices (ECDs), a flexible EC film could be integrated into a back injection molding process, where the films are processed into compact 3D geometries in a single automized step at a low processing time. Polycarbonate (PC) as a substrate is a lightweight and robust alternative to glass due to its outstanding optical and mechanical properties. In this study, an EC film on a PC substrate was fabricated and characterized for the first time. To achieve a highly transmissive and colorless bright state, poly(3,4-ethylenedioxythiophene) polystyrene sulfonate (PEDOT:PSS) was used as the working electrode, while titanium dioxide (TiO_2_) was used as the counter electrode material. They were deposited onto ITO-coated PC films using dip- and slot-die coating, respectively. The electrodes were optically and electrochemically characterized. An ECD with a polyurethane containing gel electrolyte was investigated with regard to optical properties, switching speed and cycling behavior. The ECD exhibits a color-neutral and highly transmissive bright state with a visible light transmittance of 74% and a bluish-colored state of 64%, a fast switching speed (7 s/4 s for bleaching/coloring) and a moderately stable cycling behavior over 500 cycles with a decrease in transmittance change from 10%to 7%.

## 1. Introduction

Characteristic for chromogenic systems is the fact that their optical properties can be reversibly altered by external stimuli, which make them particularly interesting in the area of architectural and automotive glazing for heat and light regulation. A classification can be made on the basis of the stimuli which can be light or UV radiation (photochromic), temperature (thermochromic), gases (gasochromic) or an electrical voltage/current (electrochromic, EC). Systems based on the latter two have the advantage to allow the active controlling of optical properties, whereas systems that respond to temperature and solar irradiation depend on external environmental conditions [1,2,3]. There are three different cell configurations for electrochromic devices (ECDs). A distinction is made between battery-like, solution-based and hybrid configurations, whereas all of them have optically transparent electrodes as current collector layers [4]. In solution-based and hybrid configurations, one or both EC materials are dissolved in a gel or liquid electrolyte and a steady current is necessary in order to maintain the colored state. In contrast, battery-like systems have an open-circuit memory due to an electrically insulating electrolyte that separates the electrodes [5]. Battery-like EC systems, i.e., multi-layer systems, might be particularly suitable for integration into plastics processing technology as the individual layers can be designed as solid structures (“all-solid-state”) that are advantageous in terms of mechanical stability, prevention of leakage and deformability [6]. The cell structure usually contains two half cells based on a substrate coated with a transparent conductive oxide (TCO) layer, e.g., indium tin oxide (ITO). Half cell 1 consists of the EC material (working electrode), while half cell 2 contains an ion storage layer or second complementarily coloring EC layer (counter electrode). An ion-conducting but electrically insulating electrolyte is sandwiched between the two half cells and can be in liquid, gel or solid form. Currently, solid polymer electrolytes are intensively investigated, in particular for applications in lithium-ion batteries. This is due to the advantages, such as mechanical stability, leakage prevention, bendability, low weight and good adhesion properties, combined with the fact that these are non-flammable in comparison to liquid organic electrolytes [7,8,9]. Among others, polyurethane as a matrix material is investigated due to its hard and soft fractions in the polymer chains, which is advantageous in terms of mechanical stability (hard segments) and ionic conductivity due to movable chain segments (soft fractions) [10,11,12]. The functionality of the EC system, that is in principle a two-electrode electrochemical cell, is based on redox reactions. When an electrical DC voltage or current is applied, cations (e.g., lithium ions) contained in the electrolyte, which are usually obtained by the dissociation of added salts, are inserted into the EC layer. In order to balance the charge, a corresponding number of electrons also migrate via the outer circuit from the counter electrode into the layer (reduction). By changing the redox states of the EC layers, the optical properties (transmittance and/or reflection) of the ECDs can be adjusted. The oxidation reaction accordingly takes place at the counter electrode, i.e., electrons are released and conducted via the outer circuit to the working electrode. When the polarity of the electrical voltage is reversed, the cations migrate from the EC layer through the electrolyte into the ion storage layer. The electron balance and thus the optical properties of the EC material returns to its original state. The ion storage layer can also have EC properties and should have a complementary redox behavior, i.e., amplify the optical change; therefore, a distinction is made between anodic and cathodic EC materials. EC materials can be categorized into five types: metal oxides (e.g., WO_3_, TiO_2_, V_2_O_5_) [13], where WO_3_ is the most widely used; metal complexes, e.g., Fe_4_[Fe(CN)_6_]_3_ known as Prussian blue [14]; metal coordination polymers [15,16]; viologens [17] and conducting conjugated polymers (e.g., polythiophenes, polyaniline, polypyrrole) [18,19,20]. Conjugated electrochromic polymers are known for their wide range of vivid colors that can be achieved by structural modifications. They have a very high coloration efficiency and high optical contrast [21,22]. Due to extensive research, its high conductivity and stability as well as commercial availability, the polymer poly(3,4-ethylenedioxythiophene) (PEDOT), often used in a polystyrene sulfonate complex compound (PEDOT:PSS), has obtained a special significance which is reflected in various applications, for example, in photovoltaics, sensing or transparent electrodes [23]. The ion storage layer should primarily have good insertion and de-insertion properties in order to store the ions and release them upon voltage reversal. This exchange significantly influences the switching kinetics of the EC cell and can influence its cycling stability [24].

To date, commercially available applications are mainly glass-based systems. In the field of architectural glazing, products are offered by SageGlass (Saint-Gobain), View Inc. and Halio Inc. Furthermore, the companies Gentex Corporation and Magna International Inc. use glass-based automatic darkening EC rear-view mirrors. Together with PPG Aerospace, a subsidiary of the artificial glass manufacturer PPG Industries, Gentex manufactures EC cabin windows for the Boeing Dreamliner. ChromoGenics manufactures commercially available EC systems based on plastic substrates. The substrate is a polyester film with inorganic EC materials (WO_3_ and NiO). Conventional windows with applied films are sold branded as “Converlight Dynamic”. 

Flexible electrochromic devices are interesting for various applications, e.g., wearables, displays, smart labels, stretchable sensors or adaptive camouflage [6,25,26,27,28,29]. The scale-up, low-cost and high-throughput manufacturing of ECDs are challenging but important for most of these applications [30,31,32]. Therefore, it must be taken into account when developing new products in these application areas. As an example, mechanically flexible and low-weight ECDs on the basis of modified PEDOT and Prussian blue as complementary EC materials were prepared by roll-to-roll processes [25]. These ECDs show advanced properties with a visible light transmittance (τ_v_) of 3% and 50% in the dark and bright state, respectively, and exhibit a switching time of <10 s (DIN A3 size) and a high cycling stability. 

The integration/processing of EC films in the common plastic (back-)injection molding process, also known as injection labeling, in order to obtain compact and 3D-formed EC plastic panes, could be advantageous with regard to the possible range of applications and mass production of ECDs (e.g., ski goggles, visors, car windows). It would be a one-step manufacturing process that could be easily automized with a low cycle time. This process is not feasible with glass as a substrate. In addition, applying EC films subsequently to more complex geometries compared to flat glass panes will be difficult due to its rigidity. Lightweight windows are especially of interest in the transportation sector. Most optical applications based on amorph plastics, such as windows and headlight coverings, are made of polycarbonate (PC) or polymethylmethacrylate (PMMA) (e.g., the well-known brands Makrolon^®^ and Plexiglas^®^). PC as substrate material in ECDs could particularly be advantageous due to its optical and mechanical properties compared to the commonly used PET. For example, the impact strength as well as haze and lightfastness are significantly better. The heat deflection temperature of 120 °C is also much higher than that of amorphous PET at 65 °C (HDT/A) [26]. So far, solid-state ECDs on PC were not yet described in the literature, only with liquid electrolyte in laboratory scale [27,28]. The goal of this study is to manufacture a battery-type, solid-state ECD with good optical properties, especially a neutral colored and highly transmissive bright state, based on PC as a substrate. For this purpose, an ion storage layer of TiO_2_ was used, for which a neutral-color-switching behavior was expected, i.e., no color change occurs at the applied voltages and the layer only serves as an ion storage layer. The above mentioned PEDOT:PSS was used as the working electrode due to its availability, ease of coating and good EC properties. Both working and counter electrodes were first characterized electrochemically. Subsequently, an ECD with a polyurethane-based gel electrolyte [29] was fabricated and characterized. This electrolyte has good optical and mechanical properties due to the cross-linked matrix, which are advantageous for the further processing of the ECD.

## 2. Materials and Methods

### 2.1. Materials

Polycarbonate films (Makrofol^®^) with a thickness of 500 µm and a visible transmittance of 91% were used as substrate (Covestro, Leverkusen, Germany). ITO, branded ‘Elamet Trans B’, was sputtered on the substrate sheets (DIN A3) by Nanogate GfO Systems GmbH (Schwäbisch Gmünd, Germany) and had a measured average sheet resistance of 22.6 ± 0.37 Ω/sq and a visible transmittance of 83%, including PC substrate. The properties of the ITO layer at around 400 nm layer thickness are comparable to those described by Laurentie et al. [28], where radiofrequency magnetron sputtering was used as a room-temperature deposition technique on PC substrate, even if slight improvements of sheet resistance and visible transmittance under optimized deposition conditions might be possible. Titanium oxide (TiO_2_, sheet resistance: >1 × 10^5^ Ω/sq) was used as ion storage layer and was coated on the PC/ITO sheets in a sol-gel process, described below. As electrochromic material PEDOT:PSS was applied as an aqueous dispersion (Clevios F AS, Heraeus, Hanau, Germany). The manufacturing process and characterization of the polymer electrolyte used in this study is described elsewhere [29]. It consists of a wide-meshed cross-linked polyurethane (PUR) matrix to which the conductive salt bis(trifluoromethane)sulfonimide lithium (LiTFSI) and the solvent propylene carbonate, both from Sigma Aldrich (St. Louis, MO, USA), were added. The polyurethane matrix was synthesized from the trifunctional polyol Desmophen 28HS98 (OH component) and the diisocyanate Desmodur XP 2617 (NCO component) (both Covestro, Leverkusen, Germany). Dibutyltin dilaurate (DBTL) from Merck KGaA (Darmstadt, Germany) was used as a reaction accelerator. The electrolyte was applied in a formulation with an sub-stoichiometric ratio of 0.85, 5 wt% salt and 10 wt% solvent, which has an ionic conductivity of about 3 × 10^−7^ S cm^−1^ [29].

### 2.2. Working Electrode: PEDOT:PSS

The PC/ITO film was cut into 5 cm wide strips and coated with PEDOT:PSS using dip coating (withdrawal speed 0.1 m min^−1^). Before the deposition of PEDOT:PSS, the ITO surface was subjected to ozone-forming UV pretreatment with a wavelength of 185 nm and 254 nm to improve dispersion spreading and thus the coating result. A distance of 2 cm and a duration of 2 min was chosen. In order to achieve a reproducible coating result, the dipping process was carried out via a servo axis with a defined speed profile. For drying and storage, the sample was annealed in an oven at 50 °C for 20 min.

### 2.3. Counter Electrode: TiO_2_

**Figure A1:**
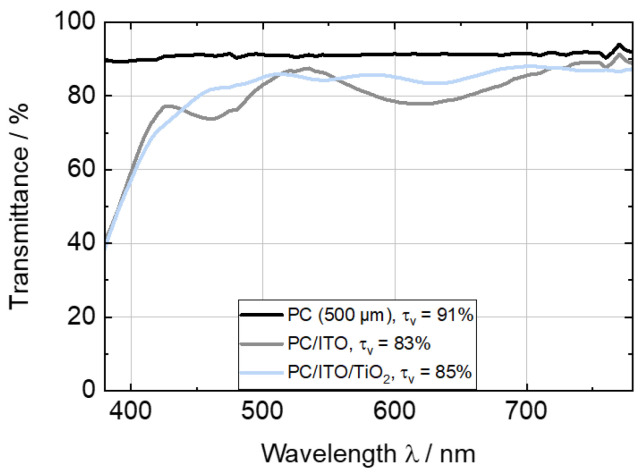
Transmittance spectra of PC substrate (500 µm), PC/ITO and counter electrode PC/ITO/TiO_2_ as prepared.

The TiO_2_ films on the PC/ITO substrates were prepared in a batch process using slot-die deposition (coating width: 250 mm). The conductive substrates were subjected to a corona pretreatment (0.15 kW). The TiO_2_ solution (7.5 wt.%) was applied at a web speed of 1.0 m min^−1^ with a delivery volume of 3.2 mL min^−1^. The distance between the slot-die and the substrate was set to 0.2 mm. The wet films (approx. 13 µm thickness) were dried at 120 °C for 10 min. The τ_v_ of the PC/ITO/TiO_2_ electrode was 85% (Figure A1).

### 2.4. ECD Assembly

The ECD consists of the working electrode (half cell 1) with the layers PC/ITO/PEDOT:PSS and the counter electrode (half cell 2) with the layers PC/ITO/TiO_2_ (manufacturing described above). The samples of each half cell were cut to a size of (5 × 2 cm²) with a scalpel. To fabricate the final ECD, two drops of electrolyte were dropped on half cell 2 with a pipette and half cell 1 was placed on top. Preliminary tests showed a more homogeneous spreading and thickness of the electrolyte layer by using a weight and bubble- or vacuole-free cells by curing under pressure. That is the reason why it was put in a pressure tank at 0.4 MPa and 40 °C for 22 h with a weight of 100 g on top in order to cure the electrolyte.

### 2.5. Instrumentation

A Solartron Metrology Ltd. (Leicester, UK) Multistat 1470E multi-channel potentiostat/galvanostat was used for the electrochemical characterization of the PEDOT:PSS half cells and the counter electrodes with TiO_2_ as well as for the ECD. All measurements were performed under argon atmosphere at room temperature with lithium as counter and reference electrode in a 1 M LiTFSI/propylene carbonate electrolyte. The cycling stability was measured using a galvanostatic charging/discharging measurement procedure for up to 1000 switching cycles (current density: 50 µA cm^−2^).

Optical, colorimetric (CIELAB color space, with L* (lightness from black to white (0–100)), a* (negative values indicate green, positive values indicate red) and b* (negative values indicate blue, positive values indicate yellow)) and spectroelectrochemical characterizations of the PEDOT:PSS half cells and counter electrodes with TiO_2_ were conducted in situ using an Avantes AvaSpec-2048 (Apeldoorn, Netherlands) standard fiber optic spectrometer combined with a balanced deuterium–halogen light source. All measurements were performed under argon atmosphere at room temperature with lithium as counter and reference electrode in a 1 M LiTFSI/propylene carbonate electrolyte. The τ_v_ was determined according to DIN EN 410 and the color was calculated according to the CIELAB nomenclature. 

A resistivity meter Loresta AX MCP-T370 from Mitsubishi Chemical Analytech Co., Ltd. (Kanagawa, Japan) was used to measure the sheet resistance. The cross-sectional images of the coated substrates to determine the film thicknesses were taken with a NVision 40 High End Cross Beam from Carl Zeiss AG (Oberkochen, Germany) consisting of two columns combining a focused ion beam (FIB) and a scanning electron microscope (SEM). Trenches have been etched into the samples by the Ga-ion beam and polished on one side to surfaces with ultralow roughness. The latter ones were sputtered with platinum and imaged via the electron beam of the SEM via induced secondary electrons. The combination of ion beam milling and cutting as well as SEM imaging allows the sectioning of thin-film heterostructure stacks at different positions for all materials accessible by SEM.

## 3. Results and Discussion

### 3.1. PEDOT:PSS Electrode

**Figure 1:**
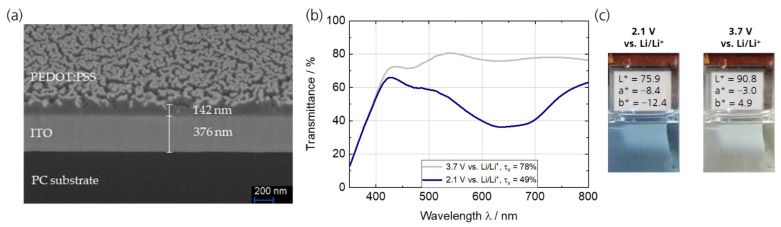
(**a**) SEM cross-sectional images of the PEDOT:PSS layer as working electrode on PC/ITO with a thickness of about 142 nm; (**b**) transmittance spectra of the PEDOT:PSS thin films on PC/ITO in 1 M LiTFSI/propylene carbonate in its charged/oxidized (3.7 V vs. Li/Li^+^) and uncharged/reduced (2.1 V vs. Li/Li^+^) state, respectively; (**c**) photographic images and L*a*b* color coordinates.

The ITO layer was about 380 nm thick (Figure 1a) and complies with the thickness in the counter electrode (Section 3.2), thereby confirming the reproducibility of the sputtering method used. Dip coating of the UV-pretreated PC/ITO films with PEDOT:PSS resulted in a dry film thickness of 142 nm. The platinum sputtered surface of the sample can be seen in the upper area of the SEM image since the image was not taken perpendicular to the specimen cross-section, but with observation angle > 0° due to preparation method. The spectroelectrochemical characterization of the PEDOT:PSS working electrode (OCP of 2.74 V vs. Li/Li^+^) is depicted in Figure 1b,c. The transmittance spectra illustrate that the bright (oxidized) state is reached at a potential of 3.7 V vs. Li/Li^+^. The PEDOT backbone is charged/oxidized and shows a weak absorption. The τ_v_ is 78%. At a potential of 2.1 V vs. Li/Li^+^, a broad absorption with a maximum at around 635 nm (τ_v_ = 49%) is responsible for the blue color that occurs when PEDOT is in its uncharged/reduced state. The photographic images and analysis of the L*a*b* color coordinates (CIELAB color space) further confirms an almost color-neutral bright state (L* = 90.8, a* = −3.0, b* = 4.9) and the blue color (L* = 75.9, a* = −8.4, b* = −12.4) of the dark state. The coloration efficiency (η = log(T_bright_/T_colored_)/q) at the absorption maximum of 635 nm is calculated to be η = 360 cm² C^−1^, which exceeds most commonly used inorganic EC materials [18] and is comparable with or exceeds PEDOT:PSS material in previous studies (e.g. on PET/ITO) [30,31]. Modified PEDOT derivatives, on the other hand, achieve significantly higher coloration efficiencies [32,33,34].

**Figure 2:**
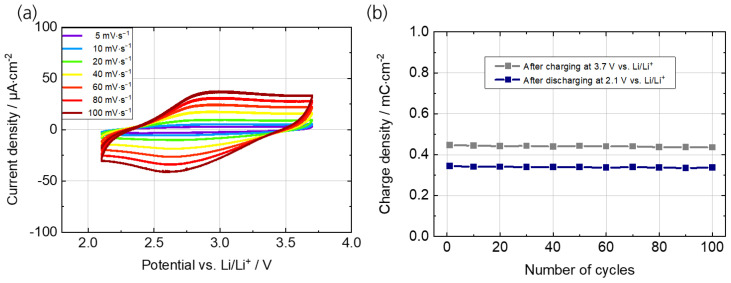
Electrochemical characterization of the PEDOT:PSS thin films on PC/ITO in 1 M LiTFSI/propylene carbonate. (**a**) Cyclic voltammograms at scan rates from 1 to 100 mV s^−1^. (**b**) Cycling stability measurements between 2.1 V and 3.7 V vs. Li/Li^+^ at a current density of 50 µA cm^−2^.

**Figure A2:**
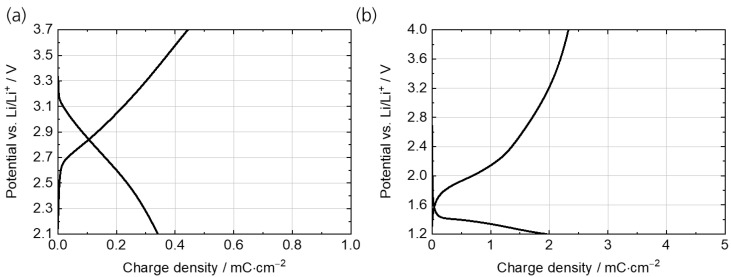
Charging/discharging curves of (**a**) PEDOT:PSS and (**b**) TiO_2_ thin films on PC/ITO at a current density of 50 µA cm^−2^.

The electrochemical properties of the PEDOT:PSS working electrode were investigated by CV and galvanostatic charging/discharging experiments over 100 cycles. Figure 2a shows the CVs at scan rates ranging from 5 to 100 mV s^−1^. The polymer thin film exhibits reversible redox peaks at a potential of 3.0 V (anodic scan) and 2.4 V (cathodic scan) vs. Li/Li^+^ (ΔE_p_ = 600 mV) corresponding to the oxidation (charging) and reduction (discharging) process, respectively, of the conjugated PEDOT backbone. The results from the CV measurements are in agreement with previously published data of PEDOT and PEDOT derivatives [35,36]. The results from the cycling stability measurements are shown in Figure 2b. The measurement was performed by charging and discharging the PEDOT:PSS thin film with a current density of 50 µA cm^−2^ (Figure A2a). The results indicate only a small decrease in charge density in the first 100 cycles (0.02 mC cm^−2^), which is equivalent to a charge retention of 88% for the charging and 85% for the discharging process. 

### 3.2. TiO_2_ Electrode

**Figure 3:**
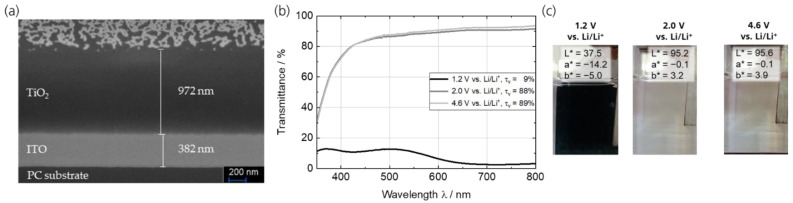
(**a**) SEM cross-sectional images of TiO_2_ ion storage layer on PC/ITO showing a porous structure and a layer thickness of about 1 µm. (**b**) Transmittance spectra of the TiO_2_ thin films on PC/ITO in 1 M LiTFSI/propylene carbonate in its charged/oxidized (4.6 V and 2.0 V vs. Li/Li^+^) and uncharged/reduced (1.2 V vs. Li/Li^+^) state, respectively. (**c**) Photographic images and L*a*b* color coordinates.

The slot-die-coated TiO_2_ layer had a film thickness of around 1 µm (Figure 3a). In the upper part of the SEM image, the platinum-coated surface can be seen, indicating the pronounced porous structure of this layer, which has already been described in [37]. The spectroelectrochemical characterization of the TiO_2_ counter electrode (OCP of 2.93 V vs. Li/Li^+^) is depicted in Figure 3b,c. The transmittance spectra illustrate that the bright (oxidized) state is reached at potentials > 2.0 V vs. Li/Li^+^. The τ_v_ is 88% (2.0 V vs. Li/Li^+^) and 89% (4.6 V vs. Li/Li^+^), respectively. At a potential of 1.2 V vs. Li/Li^+^, a broad absorption with a maximum at around 720 nm (τ_v_ = 9%) is responsible for the intensive coloration of the reduced state. The analysis of the L*a*b* values (CIELAB color space) further confirms an almost color neutral bright state (L* = 95.6, a* = −0.1, b* = 3.9) and an almost black color (L* = 37.5, a* = −14.2, b* = −5.0) of the dark state. The coloration efficiency at the absorption maximum of 720 nm is calculated to be η = 740 cm² C^−1^. Referring to the wide potential range without significant color change (2.0 V ↔ 4.6 V vs. Li/Li^+^), the cathodically coloring TiO_2_ can be used as a color-neutral counter electrode (ion storage layer) in combination with the cathodically coloring PEDOT:PSS.

**Figure 4:**
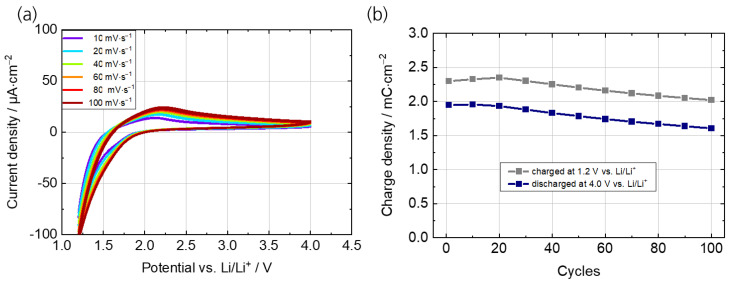
Electrochemical characterization of the TiO_2_ thin films on PC/ITO in 1 M LiTFSI/propylene carbonate. (**a**) Cyclic voltammograms at scan rates from 10 to 100 mV s^−1^. (**b**) Cycling stability measurements between 1.2 V and 4.0 V vs. Li/Li^+^ at a current density of 50 µA cm^−2^.

The electrochemical properties of the TiO_2_ counter electrode were investigated by CV and galvanostatic charging/discharging experiments over 100 cycles. Figure 4a shows the CVs at scan rates from 1 to 10 mV s^−1^. The galvanostatic charging/discharging experiments were conducted at a current density of 50 µA cm^−2^ over the course of 100 cycles (Figure A2b). The results from the cycling stability measurements (Figure 4b) indicate only a small decrease in charge density in the first 100 cycles (0.3 mC cm^−2^), which is equivalent to a charge retention of 88% for the charging and 82% for the discharging process. In particular, the CV measurements support the possibility of using TiO_2_ in the potential range from 2.0 V to 4.0 V vs. Li/Li^+^ as a color-neutral counter electrode (ion storage layer).

### 3.3. Electrochromic Device

**Figure 5:**
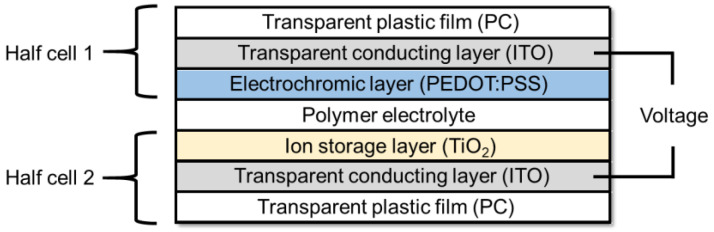
Layer structure of the battery-type ECD.

The layer structure of the manufactured battery-type ECD based on a PC/ITO substrate is depicted in Figure 5. It consists of the working electrode (half cell 1), the counter electrode (half cell 2) and a polymer gel electrolyte of around 50 µm thickness in between. The ECD was driven by a potentiostat whose clamps were connected to conductive copper tape bonded to the ITO layers of the half cells.

#### 3.3.1. Formation and Redox Behavior

**Figure 6:**
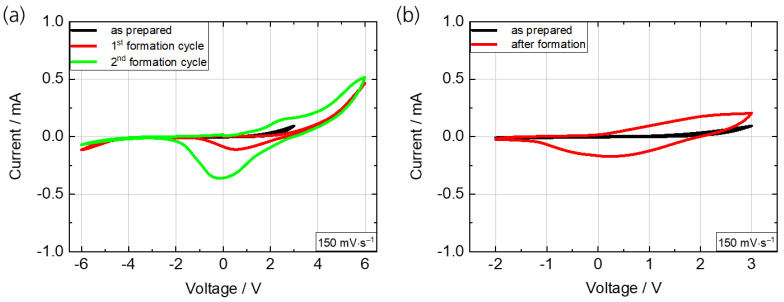
(**a**) Cyclic voltammograms of the ECD (PEDOT:PSS/TiO_2_) as prepared (−2 V/+3 V) and of two subsequent formation cycles (−/+6 V). (**b**) Cyclic voltammograms as prepared and after formation in comparison. Scan rate: 150 mV s^−1^.

The as-prepared PEDOT:PSS/TiO_2_ device shows no transmittance change when switched in a voltage range of −2 V/+3 V. It was observed that a higher initial voltage is required to activate the ECD, described as ‘formation’ in the following. Figure 6a shows the CV of the as-prepared ECD in the range of −2 V/+3 V with no redox peaks and the following two cycles of formation in a range of −6 V/+6 V starting at 0 V with clear redox peaks at around +2 V and 0 V. Considering the red curve of the first formation cycle, it is supposed that the charge density initially has to be moved in the functional layers by applying higher voltages, reflected by the current increase of up to 0.5 mA. The reduction peak occurs at around 0 V. The second cycle also shows an oxidation peak at around +2 V. These initial high voltages are not common since most of the ECDs in the literature (e.g., [19,20,25,38]) are switched in a range of maximum −/+2.5 V, because degradation processes of the electrolyte and functional layers might start or increase significantly athigher or lower voltages. However, the formation of an ECD is common in the literature [19,39]. In general, this involves the precharging/pretreatment of one EC layer before the ECD assembly. An electrolyte containing the solvent propylene carbonate with dissolved Li salt is widely used. The presented ECD is based on PC/ITO as transparent conductive substrate that is soluble in propylene carbonate. Therefore, pretreatment in such an electrolyte should lead to a swelling or even dissolution of the PC substrate. Nevertheless, it is remarkable that the devices withstand this formation step. Figure 6b shows the CVs of the ECD as prepared and after formation in comparison. Distinct anodic and cathodic peaks can be observed after formation, demonstrating a reversible redox behavior.

#### 3.3.2. Spectroelectrochemical Characterization

**Figure 7:**
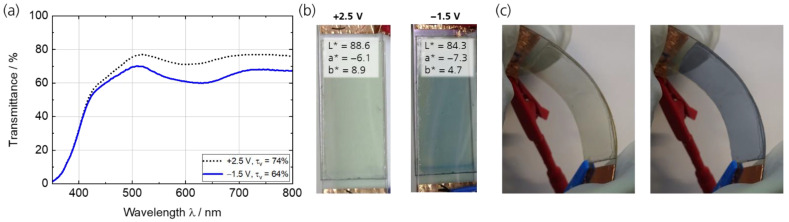
(**a**) Spectroelectrochemical characterization of the ECD (PEDOT:PSS/TiO_2_) with an active area of 2 × 4 cm² in its bright (+2.5 V) and colored state (−1.5 V); (**b**) photographic images in the bleached and colored state with L*a*b color coordinates; (**c**) a demonstration of the flexibility of the ECD in a bent condition.

The as-prepared ECD (OCV of 0.22 V) underwent formation as described in Section 3.3.1. Afterward, potentiostatic cycling with an in situ transmittance measurement was conducted. The optimal operating voltages of the ECDs were determined by a stepwise increase/decrease in the cell voltage. The bleaching/coloring voltage is the threshold voltage, at which the device is in the clear/dark state, and no further changes in the transmittance are observed at higher/lower voltages. The transmittance was measured at +2.5 V and −1.5 V and is depicted in Figure 7a for the fifth cycle with a τ_v_ in the bright state of 74% and in the colored state of 64%. Figure 7b shows the photographs and L*a*b* color coordinates of the ECD in the bright (L* = 88.6, a* = −6.1, b* = 8.9) and colored (L* = 84.3, a* = −7.3, b* = 4.7) states.

The results show that the ECD has an almost neutral color and a high τ_v_ of 74% in the bright state, but a rather small τ_v_ change of 10% compared to systems in the literature, especially compared to those with complementary switching electrochromic electrodes with an optical contrast higher than 50% [25,37,40]. A major reason for the high transmittance in the bright state might be the very thin electrode layers. 

The τ_v_ change of the ECD is considerably lower than that of the PEDOT:PSS thin films on PC/ITO (78%/49% for bright and dark state, respectively), meaning that the PEDOT:PSS is not completely reduced. It was expected that the transmittance change in the ECD with an optically passive ion storage electrode is similar to the transmittance change of the active electrode. To explain this difference, the following approaches are discussed. First, PEDOT:PSS and TiO_2_ are both cathodically coloring materials and, therefore, it is an unbalanced configuration in which TiO_2_ needs a higher charge density to obtain a properly working ECD as Hassab and Padilla described in their study [41]. Second, the precharging step may be insufficient to completely obtain a balanced ECD and thus, PEDOT:PSS switches only partially. Furthermore, the charge storage capacity of the TiO_2_ seems insufficient to completely switch the PEDOT:PSS, assuming that there is no or only a partial intercalation of Li+ ions into the TiO_2_ layer and the switching is possible due to the double-layer capacitance at the interface between the electrolyte and the TiO_2_ electrode [42]. Nevertheless, it is shown that the TiO_2_ can be used as a color-neutral counter electrode in the described potential range; therefore, ECDs with neutral color and high transmittance values in the bright state can be achieved.

#### 3.3.3. Switching Time and Cycling Stability

**Figure 8:**
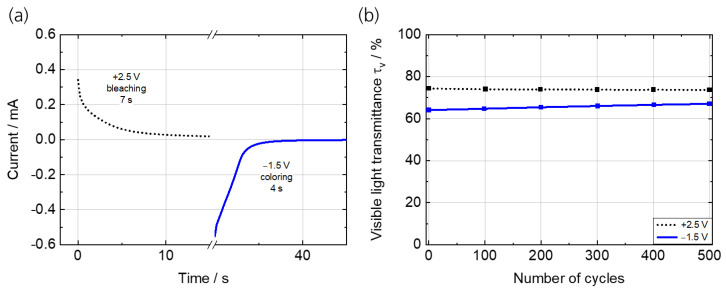
(**a**) Current–time profile of the fifth cycle with potential steps of −1.5 V and +2.5 V and a cycle time of 30 s to determine the switching time. (**b**) τ_v_ in the bright and dark state over the first 500 cycles.

**Figure A3:**
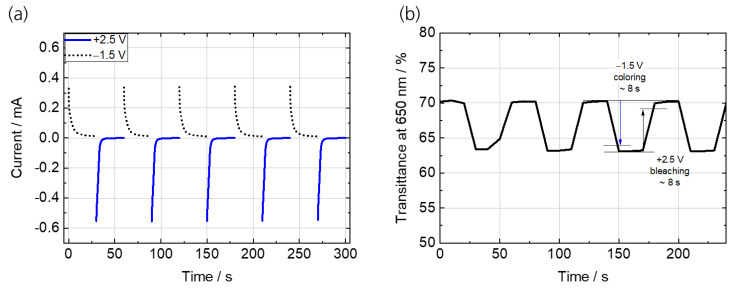
(**a**) Current–time profiles and (**b**) the transmittance over time during the first five cycles of five potentiostatic cycles of the ECD (PEDOT:PSS/TiO_2_) after the formation at cell voltages of −1.5 V (coloring) and +2.5 V (bleaching) applied for 30 s each.

Figure 8a shows the current response over time during the potentiostatic cycling with −1.5 V (for 30 s) and +2.5 V (for 30 s) (see Figure A3a for the current response and Figure A3b for the transmittance over time during the first five cycles). The switching time, defined as a current drop of 10% of the initial maximum current, is 4 s for coloring and 7 s for bleaching. The ECD was switched for 500 cycles in repeating potentiostatic cycles and the transmittance was measured in situ. The transmittance values in the bright state are constant, but the ones in the colored state logarithmically decrease (Figure 8b). The remaining transmittance change in the 500th cycle is 7%. The τ_v_ of the bright state remains constant at 74%, while τ_v_ in the dark state increases from 64% to 67%, indicating a moderately stable cycling.

**Figure A4:**
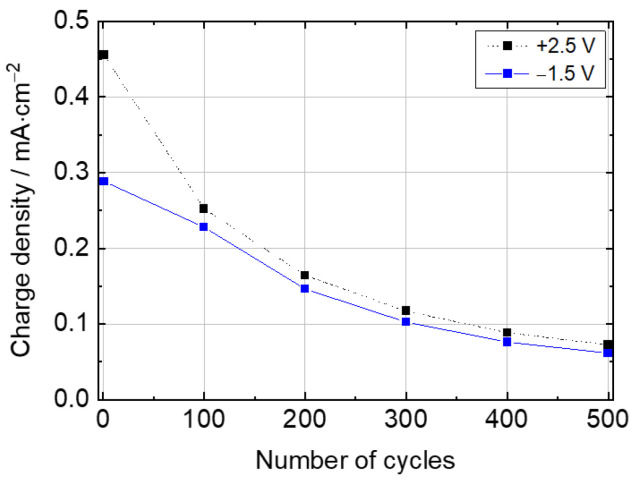
Charge density in the bright (+2.5 V) and dark state (−1.5 V) of the ECD over the course of 500 cycles.

The corresponding charge density values show a logarithmic decrease from 0.46 mC/cm² (2nd cycle) to 0.07 mC/cm² (500th cycle) at bleaching, and 0.28 mC/cm² (2 cycle) to 0.06 mC/cm² (500th cycle) at coloring (Figure A4), respectively, indicating a decrease of 85% and 79%, respectively. This evidently means that not the whole amount of charge is reversibly moved between the working and counter electrode, but some of it may possibly become adhered to each other and remains in one or both electrode layers. This phenomenon is also called ‘trapping’ in the literature [39,43,44].

## 4. Conclusions and Outlook

A flexible solid-state ECD (PEDOT:PSS/TiO_2_) with PC/ITO as the transparent conductive substrate was successfully manufactured for the first time. The PEDOT:PSS shows a high coloration efficiency (η = 360 cm² C^−1^) and a stable switching between the almost color-neutral bright state (τ_v_ = 78%, L* = 90.8, a* = −3.0, b* = 4.9) at 3.7 V vs. Li/Li^+^ and the colored state (τ_v_ = 49%, L* = 75.9, a* = −8.4, b* = −12.4) at 2.1 V vs. Li/Li^+^. The TiO_2_ shows no significant color change in the potential range of 2 V to 4.6 V vs. Li/Li^+^ and can thus be used as a color-neutral (τ_v_ = 78%, L* = 95.6, a* = −0.1, b* = 3.9) counter electrode (ion storage layer) in combination with the cathodically coloring PEDOT:PSS. The resulting ECD has a highly transmissive and color-neutral bright state (τ_v_ = 74%, L* = 88.6, a* = −6.1, b* = 8.9) and a bluish-colored dark state (τ_v_ = 64%, L* = 84.3, a* = −7.3, b* = 4.7). It shows a fast switching speed of 4 s and 7 s for coloring and bleaching, respectively, and a moderately stable cycling behavior tested for 500 cycles. A formation process of the ECD with high voltages (−/+ 6 V) is necessary. An oxidizing of the PEDOT:PSS layer as a pretreatment before ECD fabrication is known from the literature and could possibly solve this problem. An optimized cell architecture by adjusting the layer thicknesses/charge densities of the electrodes could lead to a more balanced charge exchange. To increase the τ_v_ change, especially the dark state, the charge density could be increased by modifying the TiO_2_ layer with viologens or it could be completely replaced by a complementary switching EC material such as Prussian Blue. The next step is to back-inject this type of flexible EC film by processing the films into compact 3D devices in a single step to demonstrate the feasibility of large-scale manufacturing of polycarbonate-based applications, such as ski goggles, visors and car windows.

## Data Availability

Data is available from the corresponding author on reasonable request.
